# Insights into the Transmission, Host Range, Genomics, Vaccination, and Current Epidemiology of the Monkeypox Virus

**DOI:** 10.1155/2024/8839830

**Published:** 2024-05-28

**Authors:** Yusha Araf, Jannatul Ferdous Nipa, Sabekun Naher, Sumaiya Tasnim Maliha, Hasanur Rahman, Kazi Ifthi Arafat, Mohammad Raguib Munif, Md Jamal Uddin, Nurejunnati Jeba, Sukumar Saha, Jingbo Zhai, S. M. Nazmul Hasan, Mengzhou Xue, Md. Golzar Hossain, Chunfu Zheng

**Affiliations:** ^1^Guangzhou Eighth People's Hospital, Guangzhou Medical University, Guangzhou, Guangdong, China; ^2^Department of Biotechnology, Bangladesh Agricultural University, Mymensingh 2202, Bangladesh; ^3^Department of Genetic Engineering and Biotechnology, East West University, Dhaka 1212, Bangladesh; ^4^Department of Microbiology, Faculty of Biological Sciences, University of Chittagong, Chittagong 4331, Bangladesh; ^5^Biotechnology Program, Department of Mathematics and Natural Sciences, School of Data and Sciences, BRAC University, Dhaka, Bangladesh; ^6^Department of Biotechnology and Genetic Engineering, Bangabandhu Sheikh Mujibur Rahman Science and Technology University, Faculty of Life Sciences, Gopalganj, Bangladesh; ^7^Department of Surgery and Obstetrics, Bangladesh Agricultural University, Mymensingh 2202, Bangladesh; ^8^ABEx Bio-Research Center, East Azampur, Dhaka 1230, Bangladesh; ^9^Department of Microbiology and Hygiene, Bangladesh Agricultural University, Mymensingh 2202, Bangladesh; ^10^Key Laboratory of Zoonose Prevention and Control at Universities of Inner Mongolia Autonomous Region, Medical College, Inner Mongolia Minzu University, Tongliao 028000, China; ^11^Department of Cerebrovascular Diseases, The Second Affiliated Hospital of Zhengzhou University, 2 Jingba Road, Zhengzhou, Henan 450001, China; ^12^Department of Microbiology, Immunology and Infectious Diseases, University of Calgary, Calgary, AB, Canada

## Abstract

This review delves into the historical context, current epidemiological landscape, genomics, and pathobiology of monkeypox virus (MPXV). Furthermore, it elucidates the present vaccination status and strategies to curb the spread of monkeypox. Monkeypox, caused by the *Orthopoxvirus* known as MPXV, is a zoonotic ailment. MPXV can be transmitted from person to person through respiratory droplets during prolonged face-to-face interactions. While many cases of monkeypox are self-limiting, vulnerable groups such as young children, pregnant women, and immunocompromised individuals may experience severe manifestations. Diagnosis predominantly relies on clinical presentations, complemented by laboratory techniques like RT-PCR. Although treatment is often not required, severe cases necessitate antiviral medications like tecovirimat, cidofovir, and brincidofovir. Vaccination, particularly using the smallpox vaccine, has proven instrumental in outbreak control, exhibiting an efficacy of at least 85% against mpox as evidenced by data from Africa. Mitigating transmission requires measures like wearing surgical masks, adequately covering skin lesions, and avoiding handling wild animals.

## 1. Introduction

Monkeypox, commonly referred to as “mpox,” is a reemerging zoonotic condition characterized by symptoms reminiscent of smallpox in humans and certain animals, caused by the monkeypox virus (MPXV). The World Health Organization (WHO) has recently transitioned to using “mpox” over the traditional term “monkeypox” [1, 2]. MPXV, a double-stranded DNA virus, falls within the *Orthopoxvirus* genus of the Poxviridae family, sharing this category with other human-pathogenic species such as vaccinia, variola, and cowpox [3]. While the major variola virus, responsible for Asian smallpox, boasts a fatality rate between 20% and 45%, its attenuated counterpart, variola minor or alastrim, sees rates between 1% and 2% [4]. MPXV shares close genetic and clinical ties with the variola virus; however, mpox typically presents milder symptoms than smallpox [5]. In unvaccinated populations, mpox can exhibit fatality rates up to 10%. Research conducted with about 1800 cases of mpox in the Democratic Republic of the Congo showed that individuals unvaccinated against smallpox had a 2.73- and 9.64-fold higher chance of contracting mpox in comparison to those vaccinated against smallpox. Other studies conducted in the USA (United States of America) and Spain similarly demonstrated a higher risk of contracting mpox among unvaccinated individuals compared to those who had received the smallpox vaccine [6, 7]. The virus manifests in two distinct subtypes: clade I and clade II, previously associated with the geographic domains of the Congo Basin and West Africa, respectively [8]. While both clades can induce significant illness, they often resolve without intervention. The West African clade's mortality rate stands below 4%, whereas the Congo Basin variant can escalate to 10% [9, 10]. Mpox poses specific risks to children and can lead to complications like congenital mpox or stillbirth during pregnancy [10, 11]. The disease's nomenclature stems from its 1958 discovery, following two outbreaks mimicking pox in research monkeys. The inaugural human mpox case was detected in the Democratic Republic of Congo (DRC) in 1970 [12]. Since then, human cases of mpox have been verified in multiple countries, including Cameroon, the Central African Republic, Côte d'Ivoire, Democratic Republic of Congo, Gabon, Liberia, Nigeria, Republic of the Congo, and Sierra Leone [13]. The majority of cases have been reported in the Democratic Republic of Congo [14, 15].

In 2003, the US (United States) experienced an outbreak of mpox [16, 17]. Over the past several decades, there has been a tenfold increase in the number of monkeypox (mpox) cases. Notably, the median age of affected individuals has shifted from young children, approximately 4 years old in the 1970s, to young adults with a median age of 21 years during the period from 2010 to 2019 [18]. This epidemiological shift might be attributed to the discontinuation of smallpox vaccinations, which historically provided some degree of cross-protection against mpox. Despite being identified 70 years ago, mpox has often been overlooked in medical literature, primarily due to its perception as a rare and self-resolving condition [19, 20]. On May 6, 2022, an outbreak of mpox was identified, originating from a British individual who contracted the illness during a visit to Nigeria, a country where the disease is endemic [21]. By October 31, 2022, the disease had spread to over 100 countries, resulting in 77,092 confirmed cases across nations like Australia, the UK (United Kingdom), the US, and various European countries [22]. By early September 2023, the tally of laboratory-confirmed mpox cases had risen to 89,752 [2]. Cases outside Africa have largely been linked to international travel or the importation of animals [23]. The CDC indicates that while the primary reservoir of the mpox virus remains unidentified, it is believed that African rodents and certain nonhuman primates, such as monkeys, might transmit the virus to humans [24].

The escalating number of individuals affected by MPXV necessitates an in-depth examination. This review aims to shed light on the multifaceted dimensions of the recent mpox surge. It provides an updated account of the history and present state of the MPXV disease, delving into its modes of transmission, current epidemiological landscape, and pathobiology, with a particular focus on the virus's genomic makeup. Moreover, this study presents an overview of the antiviral medications and vaccines available for the treatment and containment of MPXV infections.

## 2. History

In 1958, the renowned Danish virologist Preben von Magnus, recognized for his contributions to polio vaccines, influenza, and mpox research, identified mpox in cynomolgus monkeys within a Danish laboratory setting [12]. This discovery followed two outbreaks resembling smallpox in monkey colonies, originally from Malaysia, which were later transported to Singapore [12]. By 1964, another mpox outbreak was noted at the Rotterdam Zoo, and soon after, instances of the disease were identified in laboratory monkeys within the US [12]. After 1968, incidents of mpox in laboratory monkeys declined, attributed to enhanced living conditions for the animals and a reduced demand for monkeys from Asia and Africa, primarily used in polio vaccine production. The presence of the virus in Asian monkeys, despite the virus not being native to Asia, likely resulted from contamination or infections acquired during captivity or transit. Historically, mpox was often misdiagnosed as smallpox, chickenpox, shingles, and herpes [25]. However, as the severity and outbreak potential of mpox became evident, accurate diagnosis grew in importance [1]. The inaugural documented human case of mpox appeared in 1970, afflicting an unvaccinated infant, just nine months old, in the Équateur Province of the Democratic Republic of the Congo. Intriguingly, this occurrence was post the eradication of smallpox in the region in 1968 [26]. Since the initial identification of monkeypox (mpox), the majority of human cases have predominantly originated from the rural rainforest regions of the Congo Basin, especially in the Democratic Republic of Congo. The disease has been documented across central and west Africa. From 1970 onwards, mpox cases have emerged in 11 African countries, including Benin, Cameroon, Democratic Republic of Congo, Central African Republic, Gabon, Côte d'Ivoire, Liberia, Sierra Leone, Nigeria, Republic of Congo, and South Sudan. During the decade from 1970 to 1979, approximately 50 cases were reported, with the Democratic Republic of Congo accounting for over two-thirds of these instances [27]. Other countries, such as Liberia, Sierra Leone, the Ivory Coast, and Nigeria, also reported cases during this period [28]. Subsequently, since 1986, over 400 human cases have been recorded [28]. It is noteworthy that in tropical central and West Africa, sporadic viral outbreaks often occur, characterized by a 10% mortality rate and a 10% rate of secondary human-to-human transmission [13]. The most significant mpox outbreak was observed between February 1996 and February 1997 in these regions [12]. Outside Africa, the first significant outbreak in the US occurred in 2003. This outbreak was linked to human contact with infected pet prairie dogs, which were housed with Gambian pouched rats and dormice imported from Ghana [12]. Following an outbreak, over 70 instances of monkeypox (mpox) were recorded in the US [29]. Subsequent cases of mpox were identified in travelers from Nigeria to various countries: Israel in September 2018, Singapore in May 2019, the UK in December 2019, and the US in July and November 2021, as well as May 2021 and May 2022 [30]. Remarkably, in May 2022, mpox cases emerged in several countries where the disease was previously nonendemic [30]. The World Health Organization (WHO) data indicate that from January 2022 to September 2023, mpox was reported across 114 countries/territories/areas, with a total of 89,752 laboratory-confirmed cases. Tragically, 157 of these cases resulted in fatalities attributed to MPXV [2].

## 3. Transmission, Host, and Zoonosis

**Figure 1:**
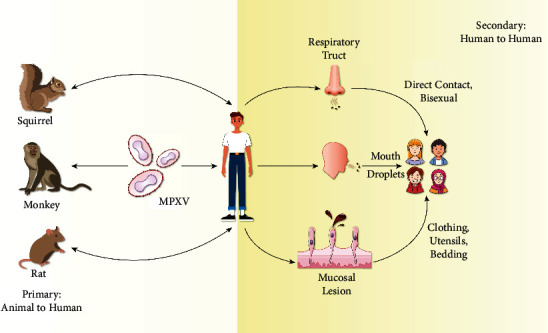
Transmission of MPXV. Primary transmission: animal-to-human by a bite, scratch, and bush meat preparation. Secondary transmission: human-to-human transmission by respiratory droplets, coughing, sneezing, and bedding.

The specified method by which MPXV spreads to humans is unknown. However, when handling MPXV-infected animals, primary animal-to-human infection is thought to happen (Figure 1) [31]. Animal-to-human (zoonotic) transmission may occur through direct (touch, bite, or scratch) or indirect contact with the blood, body fluids, cutaneous lesions, or mucosal lesions of infected animals [32]. The virus enters the body through skin breaks, some of which are not apparent, as well as the eyes, mouth, nose, and other regions of the respiratory tract. Although MPXV's reservoir host (primary disease carrier) is unknown, African rodents are thought to be involved in transmission [32]. Many African animals are infected with the MPXV, including tree squirrels, Gambian pouched rats, rope squirrels, dormice, and monkey species [32]. Eating undercooked meat and other animal-derived products from diseased animals can be a possible risk factor. People living in or close to forested areas may be indirectly or minimally exposed to infected animals [33]. The mpox outbreak in 2003 in the US began when prairie dogs in a pet shop were presumed to have been infected with the MPXV by a large Gambian rat native to Africa [34, 35]. The disease then spread through pet prairie dogs, infecting 70 people [34].

Also, secondary or human-to-human transmission is considered common [24]. Although human-to-human transmission is common, prior mpox outbreaks remained small and self-limiting because many people in the past have had some immunity (known as “cross-immunity”) from the late 20th-century smallpox mass vaccination programs. Once the at-risk population developed immunity and local herd immunity was attained, the outbreak swiftly ended. The prevalence of mpox cases in May and June 2022 points to new transmission clusters from big gatherings, such as raves and festivals [36]. Human-to-human transmission can happen when people come into close contact with infected persons' skin sores, respiratory secretions, or recently contaminated objects [24]. Also, it can be transferred by touching a mpox-infected patient's clothing, bedding, or towels and through coughs and sneezes [37, 38]. Large respiratory droplets are assumed to be the primary method of human-to-human transmission [39]. And after transmission from infected animals or humans, mpox infection begins with dermal or respiratory epithelial infection. Recently, prolonged mpox DNA shedding from the upper respiratory tract following skin lesion resolution has been observed. Therefore, there is a risk of mpox infection for those who are in close proximity to the patient during an aerosol-generating procedure (such as intubation) and are not using a surgical face mask or respirator [40]. Long-term face-to-face contact is required since respiratory droplets can only travel a few feet [38]. However, in the past few years, the number of person-to-person infections in the longest-known chain of transmission in a community increased from six to nine [41], which could be related to the loss of immunity in all groups due to the end of smallpox vaccination. Also, transmission can happen by the placenta from the mother to the fetus (might cause congenital mpox) or between and after birth through intimate contact. There have also been reports of transmission through tattoos, piercings, and sharp instruments [42]. Paul Hunter, a professor of medicine at the University of East Anglia's Norwich School of Medicine, said that “Transmission of monkeypox seems to be almost exclusively being transmitted by close and intimate contact. So, people should avoid contact with people who could be infected, especially if they have a rash. Also, the outbreak in the Democratic Republic of Congo showed that the virus circulated quickly through households [41]. The mpox is generally not a sexually transmitted disease, yet it can be transferred by direct touch during sex [43]. The MSM community (men who have sex with men) is most affected by the current mpox outbreak [43], which does not indicate that we are dealing with a new mpox strain that only affects men or is sexually transmitted between men [44]. World Health Organization (WHO) also stated that everyone with intimate contact with an infectious person is at risk of contracting mpox [44]. Recently, in the enzootic areas, mpox was detected in teenagers too. And according to WHO, serious mpox cases were found in children and are correlated with the level of virus interaction [45].

This outbreak was the first documented occurrence of mpox community transmission outside of Africa and the first known case of transmission between men who have sex with men (MSM) [44]. Before the outbreak in 2022, mpox was not considered an STD (sexually transmitted disease) [41]. The virus's rapid spread between sexual partners in the initial stages of the outbreak led to speculation that sexual intercourse could be a different mode of transmission [43]. According to epidemiologist David Heymann, mpox was likely to spread sexually among MSM in two waves in Spain and Belgium in 2022, which started the European outbreak [43]. Additionally, the CDC reevaluated recent outbreaks for potential transmissions, including kissing, hugging, oral, anal, and vaginal sex. These transmissions may be connected to genetic modifications facilitating the MPXV's human-to-human transmission [46]. The primary risk factors for monkeypox virus (MPXV) transmission are summarized in Table 1.

## 4. Genomics

**Figure 2:**
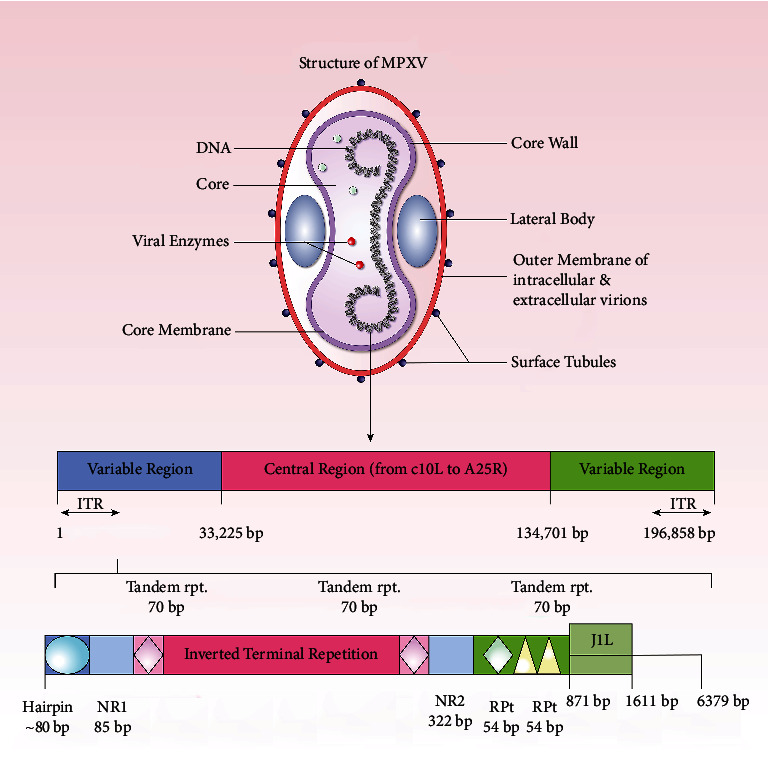
Viral and genomic structure of MPXV. The structure of MPXV shows the inside components of an mpox virus. The genomic structure shows the regions inside its DNA (MPXV: monkeypox virus and ITR: inverted terminal repeat).

The monkeypox virus (MPXV or MPV) belongs to the Poxviridae family, the Chordopoxvirinae subfamily, and the *Orthopoxvirus* genus [47]. The MPXV seems huge under electron microscopy (200–250 nanometers) [48]. Poxviruses are brick-shaped, and a lipoprotein envelope surrounds their linear double-stranded DNA genome [48]. Poxviruses have all the necessary assembly, replication, transcription, and egress proteins in their genome despite their mRNA translation's dependency on host ribosomes [47–49]. MPXV, cowpox virus (CPXV), variola virus (VARV), and vaccinia virus are among the many viruses in the Poxviridae family, *Orthopoxvirus* genus that have been observed to infect humans. Although genetically and antigenically similar, orthopoxviruses vary in host range and pathogenicity features. According to comparative genomics studies, *Orthopoxvirus* evolution is proceeding and can be influenced by a host species' selection pressure [12, 26, 50–52]. It has been suggested that the evolution of these viruses was driven by progressive gene loss, particularly toward the ends of the genome [53]. MPXV has a 197 kb linear DNA genome with 190 nonoverlapping ORFs greater than 180 nt. Like all orthopoxviruses, MPXV's main coding region sequence (CRS) at nucleotide positions 56,000–120,000 is highly conserved and surrounded by varying ends and inverted terminal repeats (ITRs) (Figure 2) [53]. Most VACV homologs of genes discovered in the MPXV genome's terminal ends are engaged in immunomodulation, and the majority are expected or confirmed to impact pathogenicity and host range determination [54].

In the ITR region of MPXV, there are at least four ORFs. MPXV strain evolution is influenced by genomic instability and genetic polymorphism [54]. Although host transition models predict genomic alterations, the relationship between gene loss and secondary transmission raises the possibility that it is evolving for effective replication in a novel ecological niche: humans [55–57].

Increased human-to-human transmission and the frequency of variation introduction could also be explained by some factors, such as vaccination status and human encroachment on reservoir habitats, linking OMCP gene loss and transmissibility coincidentally. The virus is divided into two genetic clades: clade I (Congo Basin) and clade II (West African) [58]. Observing the genomic sequences of MPXV, derived from Congo Basin and West African strains, revealed 99% identity with geographical areas and 95% identity with geographical clusters [59]. The genetic sequence of the mpox virus from an infected Portuguese man was identified in the current investigation. On May 4, 2022, a swab sample was taken from the patient's skin lesions [47]. According to phylogenetic analyses, the sequence belonged to the West African clade [53]. It was observed that the virus was the most closely connected to viruses linked to past Nigerian exports to other nations in 2018 and 2019. The reference sequence is approximately 92 percent of the length of the draft sequence [53]. However, according to WHO, no record exists that the MPXV has mutated [60]. A single nucleotide variation was found between MPXV-USA-2003-039 (human) and MPXV-USA-2003-044 (prairie dog) viruses, originating from a similar source [59, 61].

A group of closely related, large, enclosed DNA viruses called poxviruses only replicate inside the cytoplasm of vertebrate or invertebrate cells [62]. The *Orthopoxvirus* genus, part of the Poxviridae family, contains numerous zoonotic viruses, including variola, vaccinia, cowpox, and mpox [63]. The replication cycle of poxviruses provides information about how the mpox virus reproduces [64]. Poxviruses primarily rely on virus-encoded proteins to replicate in the cytoplasm. The linear, double-stranded genome (DNA) and the virus-encoded proteins and enzymes that enable transcription of the initial set of genes are located in the infectious poxvirus's membrane-bound particle's core. Initial mRNA and protein synthesis is followed by DNA replication [65]. The late and intermediate classes of mRNA can be produced using the duplicated DNA as a template. According to the most recent findings, there are only 118 early, 53 intermediate, and 38 late genes in the poxvirus [66]. The viral assembly process follows late gene expression, termed mature virions, the earliest infectious form (MV) [67].

An extracellular virion is also released when the outer and plasma membranes merge (EV) [68]. No matter the mechanism or whether the EV or MV drives infection, the fusion relies on 11-12 nonglycosylated transmembrane proteins with sizes varying between 4 and 43 kDa linked together in a complex [64]. EVs have a loose outer membrane and are specialized for leaving the intact cell and spreading inside the host, while MVs have a relatively stable outer membrane. They are hypothesized to facilitate transmission among host animals [64].

**Figure 3:**
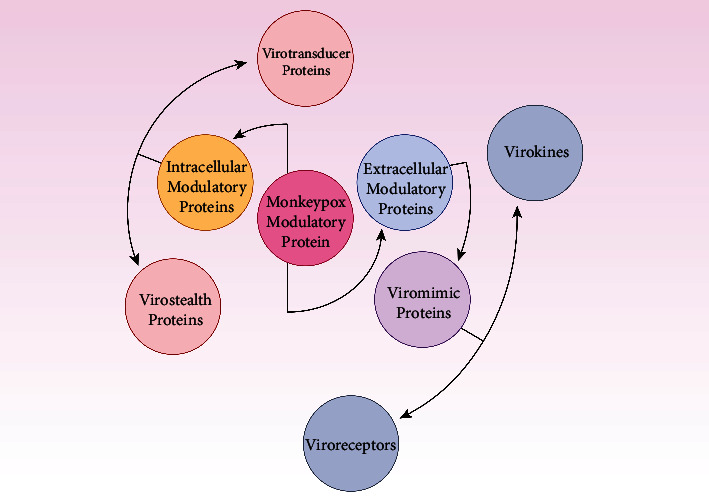
Overview of MPXV modulatory proteins. Virostealth and virotransducer proteins prevent cells from responding to monkeypox modulatory proteins and cause failure in viral detection.

Virostealth and virotransducer proteins are intracellular proteins (Figure 3). Poxvirus proteins have virostealth and virotransducer activity [69, 70]. Virotransducer proteins, especially those involved in oxidative bursts and apoptotic pathways, inhibit the cell's response to the specific infection. Downregulating the immune recognition molecules, like the MHC 1 (major histocompatibility complex class 1) and CD4^+^ by the virostealth proteins, which have intracellular functions, lowers the host's immune system to detect viruses.

**Figure 4:**
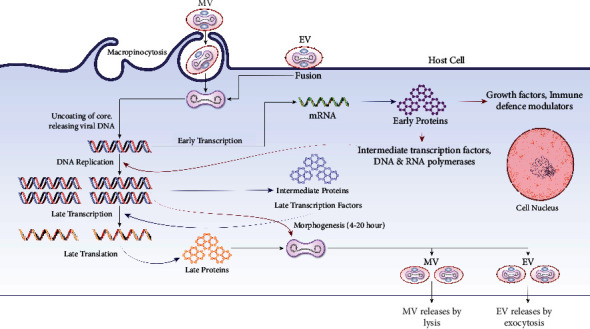
Replication of MPXV mature virion (MV). This illustration shows that the MV uses micropinocytosis and EV uses fusion to enter the host cell and the way they replicate their DNA by evading the host immune system (MV: mature virion and EV: enveloped virion).

Respiratory and other RNA viruses can escape the host's innate immune defense system [71]. Extracellular viromimic proteins fall into two categories, and both could regulate how the immune system reacts. Some viromimic proteins act extracellularly and regulate immune responses [72]. The host cytokines and chemokines are competitively bound by the baroreceptors, which are produced or present on the cell surface and inhibit the function of the host's cytokines and chemokines. Therefore, virokines create viral mimics of host growth factors, cytokines, and chemokines that efficiently abolish host immune responses against viral survival and promote viral replication and dissemination. These modulatory proteins (poxviral B22 family proteins) cooperate to circumvent and evade the host immune system (Figure 4) [73].

## 5. Pathobiology

Human mpox has a pathogenesis similar to smallpox, except that small skin lesions or oral mucous membranes are the most common routes for viral introduction from a wildlife source [74]. In extremely rare cases of person-to-person transmission, viruses can also enter through the respiratory tract [75]. The MPXV replicates in lymphoid tissue the same way as the smallpox virus but results in greater lymphadenopathy [76]. The virus first expresses itself in mononuclear phagocytic cells and then spreads via the bloodstream before reappearing in the skin cells [75, 76]. The current incubation period of mpox is up to 21 days, with a mean incubation period of 8.5 days [77].

In contrast, data from historical mpox outbreaks suggest the normal incubation period for a virus is 7 to 14 days, with a maximum of 21 days [78]. The mpox is caused by an infection of the dermis following transmission from an infected animal or an infection of the respiratory epithelium following transmission from an infected person [76]. The MPXV reproduces at the inoculation site after entering via any route, e.g., oropharynx, nasopharynx, or intradermal, and the viral entry is facilitated by the cell membrane fusion, micropinocytosis, and viral endocytosis processes [1, 79]. MPXV travels to the blood, local lymph nodes, tonsils, and other organs [1, 61].

**Figure 5:**
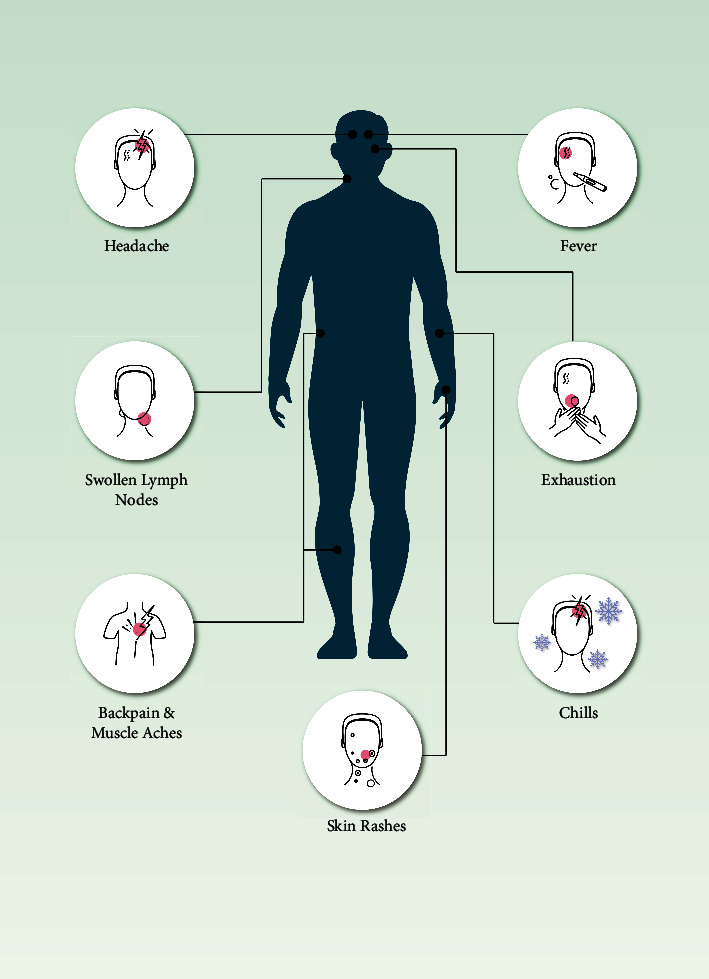
Clinical symptoms of MPXV infection. Primary and secondary viremia causes some symptoms by which MPXV can be identified.

Primary viremia and systemic infection ensue from the virus's spread through the lymphatic system [80]. Symptom onset is linked to secondary viremia, which causes prodromal symptoms, including fever and lymphadenopathy, for one to two days before lesions emerge [49]. At this time, infected patients may spread the virus. The lesions begin from the oropharynx and progress to the skin [49]. Around the time lesions appear, serum antibodies are frequently found. The second viremia causes epithelial infection, resulting in skin and mucosal sores (Figure 5) [49]. Like other poxviruses, mpox has developed methods to avoid host immune responses [24]. Fever, weariness, headache, myalgia, and lymphadenopathy are the initial signs of mpox, distinguishing it from smallpox (Figure 5). The genital area might be an initial infection site for causing rash before progressing to subsequent lesions [81]. Recent data showed that, in 95% of the mpox cases, lesions appear on the face. And in 75% of the confirmed cases, the palms, soles, and oral mucous membranes are affected. However, for genitalia and conjunctivae, it is 30% and 20%, respectively [82]. Mucosal lesions in the mouth emerge 1 to 2 days later, followed by skin lesions on the face and extremities (mainly the palms and soles), which are centrifugally condensed [14, 83–89]. The rash may or may not transmit to other body parts, and the lesions can range from a few to thousands of numbers [90]. The lesions progress via vesicular, macular, papular, and pustular phases during the next two to four weeks in one- to two-day increments [90]. Lesions are hard, deep-seated, 2 to 10 mm in size, and alter synchronously [49]. Before crusts form, lesions are in the pustular phase for five to seven days. Crusts develop and desquamate within the next seven to fourteen days, and the condition often cures three to four weeks from the symptom onset [24]. After all the crusts have fallen away, the patient is no longer regarded as contagious [24].

## 6. Current Epidemiology

Between 1980 and 1985, 282 cases were documented in Zaire at the time of initial human identification. The average fatality rate in unvaccinated cases was 11%, with higher rates in children (15%), while no mortality was found in patients who had received vaccinations [91]. After the mpox outbreak in the US in 2003, the first case of mpox this year in a nonendemic country was confirmed on May 6, 2022 [92]. A British person who had visited Lagos and Delta States in Nigeria, where mpox is regarded as an endemic disease, reported a case of mpox in early May 2022. On April 29, while in Nigeria, the person acquired a rash and traveled back to the UK, arriving on May 4 and presenting to the hospital that day [93]. The mpox was suspected quickly; thus, the patient was admitted to a hospital and isolated before testing positive for MPXV on May 6, 2022 [94]. The UK Health Security Agency (UKHSA) reported two new cases of mpox on May 12, both in London [95]. The UKHSA confirmed four more cases of mpox on May 17 in 3 Londoners and one individual from North East England (previously visited London) [96]. On the same day, the UK Health Secretary Sajid Javid announced that 11 other cases had been confirmed, which brings the total number of patients in the country to twenty [97, 98].

Through the second part of May 2022, new cases in numerous countries beyond the endemic region were recorded. Portugal reported 14 cases of mpox on May 18. Till May 18, there were seven cases confirmed in Spain [88]. The US verified its first case of mpox in 2022 on the same day, while Canada reported thirteen suspected cases. On May 19, Sweden, Belgium, and Italy confirmed their first cases. France, Australia, Germany, and the Netherlands verified their first cases on May 20 [99–101]. Several European countries, as well as Israel, verified their first cases in May. Mexico and the United Arab Emirates confirmed their first cases [102, 103]. Dr. Sue Hopkins, the UK Health Security Agency's top medical advisor, reported on May 23, 2022, that 37 new mpox cases had been verified, bringing the total number of confirmed cases in the UK to 57 [43, 104].

Beginning on May 18, cases started to be reported from an increasing number of countries and areas, especially Europe, North and South America, North Africa, Asia, and Australia [94, 105]. Following the International Health Regulations (IHRs), 780 laboratory-confirmed cases had been notified to WHO by 27 nonendemic nations in four WHO Regions as of June 2, 2022 [14].

By October 31, 2022, confirmed mpox cases had increased to 77,092, and the total number of deaths was 36 [22]. Between August and October 2022, 57 hospitalized patients with severe mpox symptoms received clinical consultation from the Centers for Disease Control and Prevention (CDC) [106]. Following the “2022 mpox outbreak” released by WHO on January 16, 2023, mpox cases were reported in 110 countries. As of January 16, 2023, there were 84,716 confirmed cases; the US had the most cases with 29,980. Since then, 80 fatalities have been reported worldwide; the top nations are the US (21), Brazil (14), Peru (12), Nigeria (7), Mexico (4), Ghana (4), Spain (3), and Cameroon (3) [8, 107]. The WHO's most recent updates indicate that the global risk of mpox is moderate. Regarding geographic distribution, the risk is highest in the Americas and moderate in Africa, the Eastern Mediterranean, Europe, and Southeast Asia [107]. From July 2023 to August 2023, 1020 new cases were reported from the United States of America, Europe, and the Western Pacific. That means a 1.2% increase is seen in total cases of mpox. Also, three new deaths were reported, all in the USA. As of 11th September 2023, among the cases with available data, 96.3% (81,352 out of 84,461) were males. The median age of these cases was 34 years, with an interquartile range of 29 to 41 years. Notably, the age and sex distributions of the cases have remained consistent over time [6].

## 7. Monkeypox in Immunocompromised Patients

Immunocompromised individuals, including those undergoing cancer treatments, organ transplants, or afflicted with autoimmune disorders, are particularly susceptible to severe manifestations of the MPXV [108]. A CDC study illustrated the graveness of this issue, showing that a significant number of hospitalized patients with MPXV were immunocompromised. These patients presented with extensive skin lesions, and a concerning 21% who were treated in intensive care units succumbed to the disease [109, 110]. The severity of MPXV in these individuals is further highlighted by two case reports: one of a patient undergoing chemotherapy for Hodgkin's lymphoma and the other a kidney transplant recipient. Both patients presented with severe symptoms, though the lymphoma patient experienced a more aggressive disease progression [111]. Despite over 80,000 globally diagnosed cases of MPXV, there is a notable absence of data from randomized controlled trials on antiviral treatments [110]. MPXV in immunocompromised patients often presents with extensive rashes, possible secondary infections, and complications like respiratory issues and organ dysfunction. These severe manifestations emphasize the need for more comprehensive research and targeted treatment approaches for this vulnerable population [108].

## 8. Vaccination

MPXV infection is usually mild; most infected people recover for a few weeks without therapy. A recent study demonstrated that vaccination did not lead to a reversal in the occurrence of mpox [112]. Different countries are already utilizing vaccines to manage mpox outbreaks [113, 114]. Currently, there are three smallpox vaccinations in the strategic national stockpile (SNS) of the US: the APSV or Aventis Pasteur smallpox vaccine could be used to treat smallpox as part of an investigational new drug (IND) protocol, along with JYNNEOSTM (also known by IMVANEX, IMVAMUNE, and MVA-BN) and ACAM2000®, which are licensed for smallpox. The JYNNEOSTM was accepted by the US Food and Drug Administration (FDA) in September 2019 and is now suggested for preventing smallpox and mpox diseases for persons of 18 years or older who have been shown to have a high risk for smallpox or mpox infection [115, 116]. JYNNEOS, a replication-deficient MVA vaccine, protects adults from developing smallpox or mpox [117]. According to historical statistics, smallpox vaccination with the vaccinia virus was around 85% effective against the MPXV [117, 118]. The CDC has an emergency access IND protocol that allows the use of ACAM2000® during an outbreak for infections caused by nonvariola orthopoxviruses (such as mpox). It was accepted by the FDA in August 2007 and replaced the earlier *Orthopoxvirus* vaccine, the Dryvax® (which the manufacturer withdrew) [119]. Between JYNNEOSTM and ACAM2000®, there are several distinctions [120–122]. ACAM2000® is a vaccinia virus capable of reproduction, whereas JYNNEOSTM is a modified vaccinia Ankara virus incapable of replication [123, 124]. Because of this, ACAM2000®, but not JYNNEOSTM, causes a significant cutaneous reaction at the inoculation site. As a result, using ACAM2000® carries a danger of accidental inoculation and autoinoculation. However, using JYNNEOSTM has no such risk. By contrasting immunologic reactions and “take” rates between ACAM2000® and Dryvax, the FDA evaluated the efficacy of ACAM2000® [125].

Similarly, the FDA evaluated the effectiveness of JYNNEOSTM by contrasting its immunologic response to that of ACAM2000® and considering supportive animal research. Avoiding ACAM2000® in populations is legitimate because recommendations state that immunosuppressed people should not use it (such as those with HIV) [126]. Additionally, the unintentional transmission that results in fetal vaccinia can be lethal to the fetus or baby and can happen when using replication-competent vaccines. Other severe adverse reactions found more commonly with ACAM2000® than with JYNNEOSTM are post-vaccination encephalitis and myopericarditis, which are estimated to affect 5.7 per 1000 main ACAM2000® vaccine recipients [125]. When approved vaccinations are unavailable or inappropriate, the replication-competent vaccinia vaccine, APSV, may be used under emergency use authorization (EUA) or an IND. Bavarian Nordic vaccines are considered safe for at-risk categories since they are made of a modified type of the vaccinia virus [109, 125].

An emerging approach to control the spread of infectious diseases like mpox is the ring vaccination strategy. This involves vaccinating immediate contacts of a confirmed infected individual, forming a protective “ring” of immunity around the infected person to break the chain of transmission. This strategy has been particularly effective in isolated communities, ensuring efficient resource utilization and limiting the spread of the disease. However, challenges arise in tracing and vaccinating certain contacts, especially in societies where there's stigma attached to behaviors such as MSM. This makes the tracing of these cases and their contacts potentially difficult, jeopardizing the effectiveness of the ring vaccination strategy. Additionally, logistical barriers in some regions and public hesitancy towards vaccination, due to potential rare adverse events, further underscore the need for comprehensive public awareness campaigns [127]. It is difficult to say whether giving the vaccination after someone has been exposed to mpox can entirely protect them because it takes between five and twenty-one days for someone who comes into close contact with an infected individual to display signs of mpox and most often, seven to fourteen days [109].

Studies indicate the acceptance of mpox vaccine's prevalence of 58.5% among healthcare workers (HCWs), with 41.5% demonstrating vaccine refusal [128]. Geographic disparities exist with Asian and African regions showing higher acceptance (68%) compared to North America and Europe (44.3%) [128]. Acceptance is notably high among physicians alone (77.1%) [128]. Higher acceptance in Asian and African regions may stem from factors like greater mpox knowledge within China due to government outreach [129–131] and potentially poorer understanding within the US compared to China [132, 133]. A similar geographic pattern existed for COVID-19 vaccine acceptance, suggesting a broader trend [134]. Cultural, religious, and local factors, coupled with rampant misinformation, all contribute to vaccine hesitancy, as seen with the COVID-19 pandemic [135–138]. Continuous monitoring and strategies to combat misinformation are crucial for improving vaccination rates [128, 139].

## 9. Antiviral Drugs

Various drugs could be used to treat mpox. One such medicine is tecovirimat, which works by interfering with a protein located on the surface of orthopoxviruses to inhibit infection from spreading [24]. Another antiviral that could be used is cidofovir, an injectable medicine approved in the UK to treat a deadly viral eye infection in people with AIDS [24]. Cidofovir is transformed into the antiviral component cidofovir diphosphate in the body [113]. Cidofovir prevents smallpox in the lab; it could be approved for use in smallpox or mpox outbreaks in the future [113]. However, cidofovir is a powerful drug that might harm the kidneys; a better choice would be brincidofovir, a similarly related drug approved in the US to treat smallpox [140]. In humans, brincidofovir has been evaluated for various viral infections [140]. Laboratory investigation proving that it acts against orthopoxviruses led to its approval for treatment in smallpox in the US [140]. As a result, brincidofovir is also included as a possible treatment for mpox. However, data on cidofovir, brincidofovir, and tecovirimat in treating human mpox infections must be included (Table 2) [140].

## 10. Approaches for Prevention and Control

While health experts believe that the concerns for the general population are low, the United Kingdom's National Health Service, the United States Centers for Disease Control and Prevention, and the World Health Organization all recommend taking certain actions to limit the risks of catching the virus [24]. When feasible, anyone with large lesions that cannot be covered easily (except facial lesions), respiratory symptoms, or draining or weeping lesions should be separated in a room away from others in the family and pets [24]. The mpox patients should stay home until they visit the doctor for follow-up care [150, 151]. Visitors must have compelling reasons to visit the doctor [150]. Members of the household who are not sick should avoid contact with the monkeypox patient [150]. Animals, especially pets, should be avoided by those with monkeypox [24]. Pets should be cared for by other household members whenever possible [24]. Patients with respiratory symptoms (e.g., cough and shortness of breath) should wear a surgical mask. If not possible (for example, if a child has monkeypox), other family members should wear a surgical mask near the monkeypox patient [152]. Disposable gloves should be worn for direct contact with lesions and discarded after that [152]. Cover up skin lesions as much as possible to reduce the danger of contact with infected persons [153].

After contact with lesion material, clothes, environmental surfaces, or linens that may have come in contact with a lesion, sick people and household contacts should disinfect their hands with soap or use an alcohol-based sanitizer. Laundry (bedding, towels, and clothing) can be cleaned using warm water and soap in a regular washing machine; bleach can be added but is not required [153]. Soiled laundry should be handled in any way that does not cause infectious particles to disperse [24]. The sick person does not need to use separate utensils if thoroughly washed. The utensils should be washed using warm water [24]. Surfaces that have been contaminated should be cleaned using some good quality disinfectants [24].

It is common for healthcare workers to come into contact with patients who have mpox. Not only mpox lesions be present in nontypical ways but also misidentifying them raises the possibility of professional encounters without the appropriate personal protective equipment (PPE). Also, exposed healthcare personnel should be kept under active supervision for 21 days to cover the incubation period. Additionally, outpatient settings should be offered in high-risk mpox transmission areas with suitable PPE, gloves, fluid-repellent surgical facemasks (FRSMs), eye protection, and FFP3 respirators for personnel [154]. Interestingly, postexposure vaccinations with the existing variola vaccines (VARVs) can be given to the exposed person. It should be given within four days to fourteen days after exposure [155]. Moreover, all healthcare personnel should get a proper idea of mpox and its symptoms through emergency training courses to increase their understanding and attitude toward mpox outbreak response and prevention [156].

## 11. Conclusions

The re-emergence of monkeypox (mpox) underscores a critical concern for global health, potentially heralding the onset of a future pandemic. Contemporary evidence suggests that a decline in human immunity may have set the stage for the resurgence of this virus. Recent detections of MPXV cases beyond Africa accentuate the potential for its geographical spread and underline the global implications of this disease. It is imperative that public health communities recognize the escalating risk posed by mpox and prioritize efforts to mitigate its spread. Healthcare practitioners attending to affected patients must exercise meticulous caution to curtail human-to-human transmission. The exigent need of the hour is the rapid development and deployment of MPXV-specific antiviral agents and vaccines. Initiating vaccination campaigns in regions with confirmed MPXV presence, particularly Africa, is paramount. Additionally, substantial international funding should be mobilized to bolster case detection and surveillance efforts globally. To navigate the evolving landscape of mpox epidemiology, rigorous research endeavors are essential. Comprehensive understanding and awareness are our foremost tools in preempting and managing potential MPXV outbreaks. Collaborative efforts, spearheaded by organizations like WHO and augmented by the influential reach of social media, can amplify global awareness. By harnessing informed strategies and fostering international collaboration, we can proactively counteract the looming threat of a MPXV-induced pandemic.

## Figures and Tables

**Table 1:** Risk factors for MPXV transmission.

Risk factor	Description	Transmission routes	References
Animal-to-human (zoonotic)	Exposure to infected animals, particularly rodents and primates in endemic areas	(i) Direct contact with bodily fluids or lesions (bites and scratches) (ii) Indirect contact with contaminated objects (bedding and so on) (iii) Consumption of undercooked meat from infected animals	[31–33]

Close contact with an infected person	Interactions with someone who has MPXV	(i) Prolonged face-to-face contact, leading to exposure to respiratory droplets (ii) Direct contact with lesions, scabs, or bodily fluids (iii) Contaminated objects or surfaces	[31, 36–39]

Sexual contact (especially among MSM)	Intimate contact with an infected person	(i) Skin-to-skin contact during sexual activity	[36, 43, 44]

Other potential risk factors	Less common or under investigation	(i) Placental transmission (mother to fetus) (ii) During or after birth through close contact (iii) Tattoos, piercings, and sharp instruments (iv) Aerosol generation during medical procedures	[40–42]

**Table 2:** A list of treatments for mpox patients.

Therapeutic agent	Tecovirimat	Brincidofovir	Cidofovir	Vaccinia immune globulin	Trifluridine
Dosing	For 14 days, adults should take 600 mg B.I.D. If a child weighs between 13 kg and 25 kg, they should take 200 mg daily. For body weight, more than 40 kg and between 25 and 40 kg, take 400 mg daily through P.O., IV (accepted in May 2022).	Adults and pediatric patients between 10 kg and 48 kg take 4 mg/kg of oral suspension once weekly; adults above 48 kg receive 200 mg twice weekly. Patients with low weights can receive oral and intravenous dosages. Pediatric patients weighing less than 10 kg are given 6 mg/kg.	Initially, 5 mg/kg IV should be administered once every two weeks for two weeks.	Based on the severity of the symptoms and the patient's reaction to therapy, 6000 U/kg may be repeated as soon as symptoms emerge; 9000 U/kg may be considered if the patient does not respond to the initial dosage.	Trifluridine is typically administered as eye drops. Initial dosing involves application at 2-hour intervals until there is complete regeneration of the corneal epithelium. Following this, it is administered once every 4 hours for another 7 days. It is noteworthy that trifluridine is not intended for prolonged use. If treatment extends beyond 3 weeks, alternative pharmacological options should be considered.

Action style	VP37 envelope-wrapping protein inhibitor for *Orthopoxvirus*.	*Orthopoxvirus* DNA polymerase-mediated viral DNA synthesis is specifically inhibited by the active metabolite, cidofovir diphosphate, which is phosphorylated to form.	Cellular phosphorylation is followed by targeted inhibition of viral DNA synthesis mediated by the *Orthopoxvirus* DNA polymerase.	Passive protection is given by the antibodies obtained from the combined human plasma of smallpox vaccination recipients.	Trifluridine acts by inhibiting DNA synthesis, targeting the enzymes responsible for this process. Furthermore, it may integrate into DNA. The drug's mechanism primarily revolves around its interference with DNA replication, thereby stunting the growth and multiplication of the mpox virus.

Side effects	Nausea, vomiting, headache, and stomach pain. IV form might result in infusion reaction sites.	Abdominal discomfort, nausea, vomiting, and diarrhea.	Low serum bicarbonate levels can cause proteinuria, neutropenia, infection, hypotony of the eye, iritis, uveitis, nephrotoxicity, and fever.	Headache, nauseous, queasy, and rigors.	Transient local burning sensation, eyelid oedema, inflammation of the cornea, and allergic reactions.

Precautions (US labeling)	None	None	History of probenecid or other sulfa-containing medications causing clinically severe hypersensitivity; hypersensitivity to cidofovir or any component of the formulation; use with or within seven days after nephrotoxic drugs; direct intraocular injection; serum creatinine >1.5 mg/dl; urine protein >100 mg/dl (>2+ proteinuria); CrCl 55 ml/min.	Isolated vaccinia keratitis, a history of anaphylaxis or a severe systemic reaction to human globulins, an IgA deficiency with IgA-blocking antibodies, and an IgA hypersensitivity history.	Should be avoided in prolonged usage to prevent corneal epithelial toxicity.

Significant medication interactions	Hypoglycemia brought on by repaglinide and midazolam's diminished effectiveness. Note: combining repaglinide with other medications may result in hypoglycemia. During co-administration, check blood sugar levels and look out for hypoglycemia symptoms.	Brincidofovir exposure is increased by OATP1B1 and 1B3 inhibitors, which may increase brincidofovir-related side effects. Examine possible substitute medications that do not contain OATP1B1 or 1B3 inhibitors.	Or nephrotoxic agents, such as probenecid.	It contains maltose, which may cause high glucose levels, resulting in untreated hypoglycemia or improper insulin dosing. It may also reduce the effectiveness of live attenuated virus vaccinations. Revaccination can be required, and some serological tests might be affected.	No specific drug interactions are mentioned.

Apply to a certain population	P.O.: no hepatic or renal correction is necessary. Patients who have significant renal impairment should not receive IV therapy.	Not suggested for expecting or nursing women (perform a pregnancy test in women of childbearing potential before treatment). As brincidofovir may result in elevations in blood transaminases and serum bilirubin, conduct liver function tests before and during therapy.	Renal function must be considered while adjusting the dose: CrCl 55 mL/minute, urine protein 100 mg/dL, or serum creatinine >1.5 mg/dL (>2+ proteinuria).	Renal function must be considered while adjusting the dose: urine protein 100 mg/dL, serum creatinine >1.5 mg/dL (>2+ proteinuria), and CrCl 55 mL/minute.	Patients with ocular manifestations due to mpox virus infection, especially those presenting with conjunctivitis, keratitis, or other ocular symptoms, are the primary candidates for these therapeutic agents. The majority of affected individuals in past outbreaks were young children (<10 years of age).

Reference	[141, 142]	[122, 143]	[122, 143]	[144, 145]	[146–149]

## Data Availability

No data were used to support this study.
